# Surgical Site Infection Following Intramedullary Nailing of Subtrochanteric Femoral Fractures

**DOI:** 10.3390/jcm10153331

**Published:** 2021-07-28

**Authors:** Michalis Panteli, James S. H. Vun, Robert M. West, Anthony Howard, Ippokratis Pountos, Peter V. Giannoudis

**Affiliations:** 1 Academic Department of Trauma & Orthopaedics, School of Medicine, University of Leeds, Leeds LS2 9LU, UK; j.vun@nhs.net (J.S.H.V.); anthonyjhoward@aol.com (A.H.); I.Pountos@leeds.ac.uk (I.P.); pgiannoudi@aol.com (P.V.G.); 2 Leeds Institute of Rheumatic and Musculoskeletal Medicine, University of Leeds, Leeds LS7 4SA, UK; 3 Leeds Orthopaedic & Trauma Sciences, Leeds General Infirmary, University of Leeds, Leeds LS2 9LU, UK; 4 NIHR Leeds Biomedical Research Centre, Chapel Allerton Hospital, Leeds LS7 4SA, UK; 5 Leeds Institute of Health Sciences, University of Leeds, Leeds LS2 9LU, UK; R.M.West@leeds.ac.uk

**Keywords:** subtrochanteric, femur, fracture(s), non-union, infection

## Abstract

**Aim:** To investigate the incidence, risk factors and pathogenic micro-organisms causing superficial and deep infection in subtrochanteric femoral fractures managed with an intramedullary nail. **Materials and Methods:** Following institutional board approval, all consecutive patients presenting with a subtrochanteric fracture were retrospectively identified, over an 8-year period. Basic demographics, fracture characteristics, fracture union, revision operation, mortality and other complications were reported and analysed. Variables deemed statistically significant (*p*-value < 0.05) were then included into a revised adjusted model of logistic regression analysis, where we reported on the odds ratio (OR). **Results:** The overall incidence of infection was 6.4% (*n* = 36/561; superficial: 3.7%; deep: 2.7%). Associations with deep infection included: non-union (OR 9.29 (2.56–3.38)), the presence of an open fracture (OR 4.23 (3.18–5.61)), the need for massive transfusion (OR 1.42 (2.39–8.39)), post-operative transfusion (OR 1.40 (1.10–1.79)) and prolonged length of stay (OR 1.04 (1.02–1.06)). The Commonest causes of superficial infection were *Staphylococcus aureus* (28.5%), enteric flora (23.8%) and mixed flora (23.8%); whereas coliforms (60%) and *Staphylococcus aureus* (26.7%) were the commonest micro-organisms isolated in deep infection. Polymicrobial infection was identified in 38.5% and 80% of superficial and deep infections, respectively. **Conclusion:** Causative micro-organisms identified in both superficial and deep infection were similar to those reported in post-traumatic osteomyelitis. In an attempt to minimise infection, the treating clinician should focus on modifiable risk factors with adequate patient optimisation, prompt surgical treatment, adequate antibiotic coverage and wound care when treating patients with subtrochanteric femur fracture.

## 1. Introduction

Subtrochanteric fractures represent a subgroup of proximal femur fractures, located between the lesser trochanter and 5 cm distal from it [1]. It is widely recognised that subtrochanteric fracture is a challenging fracture pattern to treat, due to the moderate blood supply of the subtrochanteric region [2], and the pull of opposing forces to which it is constantly subjected [3,4,5].

Surgical site infection (SSI) is one of the leading causes of healthcare-associated infections, with significant impact on both the patients and healthcare system such as prolonged admission, loss of quality of life, and increased mortality [6,7,8,9,10,11,12]. Risk factors for SSIs include patient-related factors such as advanced age, co-morbidities, cognitive impairment and smoking, as well as surgery-related factors such as delay to operation, duration of the operation and presence of intra-operative complications [13,14]. *Staphylococcus aureus* (*S. aureus*) is the predominant organism isolated from such infections, having an increasing incidence in the last few decades [15,16,17,18]. Polymicrobic infections have also been reported in as many as 29% of the cases, posing an additional challenge to their treatment, leading to a synergistic resistance to a wide range of antibiotics [17,19,20]. Interestingly, culture-negative infections have been reported in up to 32% of patients [18,19], possibly because of the early targeted start of empirical antibiotic therapy, or the presence of biofilms isolating these organisms from the environment [21,22].

Subtrochanteric fractures have been widely reported to run a high risk of developing implant-related complications and fracture non-union [23,24,25], and most importantly high rates of re-operation [26,27,28,29]. However, despite SSI being such a common complication, published studies on subtrochanteric focus were mainly focused on outcomes in terms of fracture healing and survivorship [23,24,25,26]. As such, knowledge surrounding the incidence, associations, pathogenic micro-organisms and the management outcomes of SSIs in surgically treated subtrochanteric fracture remains lacking.

The aim of this study was therefore to report on the incidence, causative organisms and treatment outcomes of both superficial and deep infection in subtrochanteric femur fractures treated surgically with an intramedullary (IM) nail. We also investigate risk factors associated with SSIs in subtrochanteric femur fractures.

## 2. Materials and Methods

This study gained approval from the regional institutional board. All consecutive patients presenting in a Level 1 Trauma Centre with a fracture involving the subtrochanteric region over an 8-year period were retrospectively identified. The inclusion criteria were skeletally mature patients presenting with a subtrochanteric fracture, managed with an IM nail. The exclusion criteria comprised patients undergoing their primary operations at other institutions and patients whose care was transferred to other institutions following their operations.

Data on basic demographics, past medical history, mechanism of injury, operation characteristics, pathogenic microorganisms isolated from conventional cultures, treatment outcomes and complications were collected from medical case notes, electronic archiving systems and theatre records. The Russell Taylor classification system was used to describe the fractures [30,31].

Surgical site infections (SSIs) were classified into superficial or deep infections based upon the depth of the tissue involved [32,33]. Superficial infections were defined as those occurring during the early post-operative period around the incision site, associated with erythema, warmth, discharge and raised inflammatory markers [33], commonly managed with short term oral antibiotic treatment and retention of metalwork. Deep infections were defined as those involving the fascial, muscular layers and beyond [32], often requiring further surgical intervention (i.e., washout, revision procedure) and prolonged intra-venous antibiotic treatment.

The diagnosis of SSI in our Trauma Centre was based upon four main criteria: (i) clinical diagnosis; (ii) serological markers for inflammation and infection; (iii) imaging and (iv) microbiological diagnosis. Clinical diagnosis included patient assessment for symptoms and signs of systemic infection (e.g., malaise, sweats, chills, fever, tachypnoea, tachycardia), and assessment of the patient’s surgical incision for signs of superficial or deep infection, as previously described.

Serological markers advantageous to aiding diagnosis include the leukocyte count (as part of FBC), CRP and ESR. These serological tests were performed in addition to other standard blood tests such as U&Es and LFTs, providing a useful assessment of the general physiological status of the patient. The most commonly used imaging techniques include plain radiographs and ultrasound. Plain radiographs were used to check for signs of deep infection such as soft tissue oedema, fluid levels, or gas in the intra/submuscular planes, and evidence of osteomyelitis and lucency surrounding the metalwork. Ultrasound is also useful in determining the fluctuance, depth and communication of any suspected collection. Furthermore, ultrasound could also find its use as an interventional procedure in the form of ultrasound-guided biopsy or drainage to further offload infection and aid diagnosis. In equivocal or quiescent cases of infection, MRI scans were performed with specialist input from a musculoskeletal specialist radiologist.

In terms of diagnosing the pathogenic micro-organisms responsible for infections, microbiology tissue swab from the surgical incision, and peripheral blood cultures were taken from all patients. These were processed by the microbiology laboratory for microscopy examination, gram staining, and extended cultures of two weeks. Additionally, for patients with deep infection requiring surgical intervention, at least 5 deep tissue samples were sent for microbiological culture, with the growth of indistinguishable organisms on 3 or more samples known to have a sensitivity level of 66% and a specificity of 99.6% [34]. None of the samples were processed for qPCR analysis as it is not routine practice in our local NHS Trust. Tests for fungal infections were only performed if clinically indicated (i.e., immunocompromised patients and/or failure of antibiotic treatment).

In terms of management, patients were started on an antibiotic regimen according to our local hospital guidelines, and always through a multidisciplinary approach including microbiology advice. More specifically, empirical antibiotics were administered until the results of the cultures were available, then changed to targeted antibiotics according to sensitivities. In cases of negative cultures, broad spectrum antibiotics were used. Surgical intervention in cases of deep infection would at least entail surgical wound washout and debridement to clear the source of infection, and to reduce the risk of biofilm formation on the metalwork and subsequent intramedullary spread/sepsis. If the depth of infection was suspected to involve the medullary canal, exchange nailing would be performed by first removing the metalwork, reaming the medullary canal to remove any potential inoculations and pockets of infection before insertion of the new IM nail. In our unit, the reamer-irrigator-aspirator (RIA) technique is the preferred option for debriding medullary canal, as it achieves both the efficient debridement and immediate evacuation of the debrided infected tissues via its aspirator function, thereby guaranteeing a more successful management of the medullary infection. Finally, a staged approach is considered when there is suspicion of severe intramedullary spread that require adjuvant antibiotic therapy to ensure adequate eradication of infection. This would entail metalwork removal, debridement of medullary canal and insertion of a cement nail as the first stage, with the new definitive IM nail performed at a later stage once the infection is adequately eradicated. The cement nail was custom made by the operating surgeon using a long intramedullary guide wire cut to the size of the intramedullary canal, with antibiotic-loaded cement applied around it to the diameter of the medullary canal, and both ends of the guide wire bent to ensure containment of the cement.

## 3. Statistical Analysis

Statistical analysis was performed using the computing environment R (R version 3.6.0) [35]. Basic demographic data were presented as count (percentage) or as mean ± SD. Following normality assessment, parametric and non-parametric data were analysed using Pearson’s chi square test and Welch unpaired independent *t*-test, respectively. We consider a *p*-value of < 0.05 as statistically significant. A simple logistic regression model was first used for the initial analysis, to identify potential unadjusted associations with deep infection. A revised adjusted model of multiple logistic regression was then developed after the stepwise removal of co-variates performed based upon their likelihood-ratio and chi-square *p*-values. Reported coefficients and OR from this revised adjusted multiple logistic regression analysis were then used to identify associations with deep infections.

## 4. Results

### 4.1. Basic Demographics

A total of 561 patients (217 male; mean age: 73.1 years old, SD: 19.1 years) with subtrochanteric femur fractures treated with an intramedullary nail were included into the study. The incidence of SSI was 6.4% (*n* = 36/561), of which 3.7% (*n* = 21) were classified as superficial infections, whilst 2.7% (*n* = 15) were classified as deep infections. Full details on basic demographics, injury characteristics, medical comorbidities, operation characteristics, radiographic measurements, complications, length of stay, recurrence of infection and mortality on patients (no infection/superficial infection/deep infection) are illustrated in Table 1. All superficial infections were observed within the first 30 days post operation (early infections). In deep infections, on the other hand, the mean time to revision was 12.2 months (SD 10.2 months) (late infections).

### 4.2. Pathogenic Micro-Organisms

The three commonest pathogenic micro-organisms isolated from superficial wound infections (*n* = 13) were *Staphylococcus aureus* (46.2%), followed by enteric flora (38.5%) and mixed skin flora (38.5%). Other less commonly isolated organisms in patients with superficial infection include Enterococcus species, Gram-negative bacillus, and *Beta Haemolytic Streptococcus*.

In patients with deep infections, the commonest micro-organisms were *Coliforms* (60.0%), followed by *Staphylococcus aureus* (26.7%). Other micro-organisms isolated from deep infection include *Proteus*, *Staphylococcus epidermidis*, *Coagulase negative staphylococcus*, *Pseudomonas aeruginosa*, Gram-negative bacillus, and *Beta Haemolytic Dermabacter hominis*. No micro-organisms were isolated on prolonged microbiological cultures in three patients (20%) with clinical and serological markers indicating deep infection.

Polymicrobial infection was not uncommon in subtrochanteric femur fracture patients treated with IM nailing, accounting for 47.2% of all cases with SSI. When categorised into superficial and infections, it was clear that polymicrobial infection accounted for the majority of the cases of deep infections (80%, *n* = 5/13), whereas it was only present in 38.5% of superficial infections (*n* = 5/13) (Table 2).

### 4.3. Outcomes

All patients with superficial infection were successfully treated according to our local hospital’s antibiotic guidelines, which, depending on severity, could be in the form of a short course of either oral or intravenous antibiotics. Intravenous antibiotics were administered in cases where a tracking cellulitis was identified as originating from the site of superficial infection.

Overall, 80% of all patients with a deep infection (*n* = 12/15 patients) underwent re-operation(s) to address the deep infection and its sequelae (number of revisions: median 3, SD 3.8). The breakdown of operation type was illustrated in Table 3. Surgical wound washout and debridement of the necrotic / infected tissue was performed on all patients, either as definitive surgery (25%, *n* = 3 /12 patients) or as part of an operation (75%). The most commonly performed revision operations include staged metalwork removal (*n* = 9), exchange nailing (*n* = 5), insertion of a cement nail (*n* = 3), local antibiotic therapy (antibiotic beads, antibiotic cement; *n* = 3) and RIA debridement of medullary canal (*n* = 4). As for the remaining 20% patients (*n* = 3), one patient died before revision surgery could occur, whereas the other two patients declined surgery and had their infection suppressed with prolonged course of intravenous and oral antibiotics.

In terms of fracture healing following deep infection, only two patients had uncomplicated fracture healing (13.3%, *n* = 2/15), whilst one patient died before revision surgery (6.7%); we observed deep infection to be an independent risk factor for fracture non-union (*p* < 0.001). Majority of patients either developed fracture non-union (66.7%, *n* = 10/15) or delayed union (13.3%, *n* = 2/15). The mortality rate of patients at a median follow up of 35.05 months follow up (IQr: 41.48 months, range: 4.10–62.80 months) was 20% (*n* = 3/15).

### 4.4. Associations with Deep Infection

Comparing the characteristics of patients developing a deep infection with those who did not (i.e., no infection or only superficial infection), several factors appeared to be associated with deep infection (Table 4). The presence of an open fracture (*p* = 0.001) and fractures extending distally toward the femoral shaft (*p* < 0.001) were the only two fracture characteristics significantly more common in patients with deep infection. In terms of patient demographics, medical comorbidities and social history, only patients with a history of increased alcohol intake were associated with a higher risk for a deep infection (*p* = 0.041). Age, gender, ISS > 16, diabetes, malignancy, steroid use, smoking history and patient mobility were not significantly different between patients with deep infection and those without. Peri-operatively, patients who required open reduction (*p* = 0.019), increased surgical time (*p* = 0.026), and increased total time of the procedure (induction to recovery; *p* < 0.001) demonstrated statistical significance. Regarding complications, presence of deep infection was associated with a higher risk of non-union (*p* < 0.001), post-operative transfusion (*p* = 0.040) and massive transfusion (*p* < 0.001), increased length of stay (LOS: *p* < 0.001) and the need for escalation of care in HDU / ICU (*p* = 0.029).

Regression analysis with adjustment of the different aforementioned variables identified progression to non-union (OR 9.29 (2.56–3.38)) and the presence of an open fracture (OR 4.23 (3.18–5.61)) as the most significant associations with deep infection. The need for massive transfusion (OR 1.42 (2.39–8.39)), post-operative transfusion (OR 1.40 (1.10–1.79)) and prolonged LOS (OR 1.04 (1.02–1.06)) were also associated with deep infections (Table 5).

## 5. Discussion

According to the Centres for Disease Control and Prevention (CDC) and World Health Organisation (WHO), SSIs are preventable in the majority of cases [36,37]. Inasmuch as recommendations and evidence-based strategies recommended by CDC and WHO are followed, SSI still remains one of the leading causes of healthcare-associated infections, with the median cost of treating SSI nearly double that of patients without infection (SSI: USD 108,782 vs. no infection: USD 57,418) [38]. Furthermore, SSI is also associated with increased morbidity and prolonged hospital admission [39].

Although subtrochanteric femur fractures are commonly encountered, the majority of the published literature is based upon a small sample size [6,7,8,9,10,11,12]. Hence, there remains a need for bigger cohort studies to allow us to better understand the incidence, pathogenic micro-organisms, outcomes and complications of treatment of SSI in patients with subtrochanteric femur fractures.

From our case series of 561 subtrochanteric fractures, we reported an incidence of SSI at 6.4% (superficial infection: 3.7%; deep infection: 2.7%); this is comparable with that reported by Kilinic et al.’s study of 52 subtrochanteric fractures treated with IM nailing and cerclage wiring [40]. The majority of other studies in the literature reported a lower incidence of SSI (0.0–1.7%), which could be secondary to the small sample size in these studies and inconsistency in their definition of infection [6,7,8,9,10,11,12].

The successful treatment of SSI depends hugely on the accurate identification of the pathogenic micro-organisms. Interestingly, there is currently a lack of published evidence on this important topic. From our cohort of patients with SSI, *Staphylococcus aureus* was found to be the commonest isolated micro-organism (superficial infections: 46.2%; deep infections: 26.7%). The incidence of *Staphylococcus aureus* SSI in subtrochanteric femur fractures is similar to that observed in patients with post-traumatic osteomyelitis, whereby the incidence range between 35–50% [17,18,19,41]. For deep infections alone, *Coliforms* were found to the commonest micro-organism (60.0%). Although the overall incidence of *Coliform* deep infections was similar to that published in the literature (*Enterobacter cloacae* 11–12%; *Klebsiella pneumoniae* 4–12%; *Citrobacter koseri* 2%) [17,18], we found the incidence of *Escherichia coli* deep infection in our cohort of subtrochanteric fractures to be nearly double that reported in the literature. The incidence of polymicrobial infection in our cohort of subtrochanteric infections (38.5% of all superficial infections; 80% of all deep infections) was much higher than that reported in post-traumatic osteomyelitis (17–29%) [17,19,41]. No organism was isolated on 20% of conventional cultures on our cohort patients with deep infection, which is comparable to the results found in the literature on post-traumatic osteomyelitis (11–32%) [18,19].

Plausible reasons for negative cultures in some of our patients include: (i) the early targeted start of broad-spectrum antibiotic therapy upon clinical suspicion of infection before obtaining deep tissue cultures; or (ii) the inability of conventional culture techniques to detect bacteria situated within a biofilm [42,43]. Taken altogether, this further highlights the complexity of deep infections in subtrochanteric fractures. Firstly, the antibiotics alone may not successfully eradicate deep infections. Secondly, it is important to consider the need for re-operation (in addition to antibiotics) early to avoid a protracted course of infection and therefore risking biofilm formation, intramedullary spread and osteomyelitis. Finally, it is equally important to collaborate with microbiologists to guarantee successful targeted antibiotic use to eradicate the infection, or at least successful suppression of infection whereby surgery was deemed unsafe or impossible. In those instances, the use of qPCR (in addition to standard gram staining and extended microbiological cultures) could be advantageous in aiding the accurate diagnosis, targeted use of antibiotics and hence minimising the risk of causing multi-drug resistant micro-organisms.

The early identification and treatment of SSIs, in particular deep infection, is crucially important given the high risk of non-union, delayed union and mortality, as highlighted by our study. Furthermore, the median number of re-operations in our cohort surgery is four operations. This in itself carries significant risk to the patient directly through re-operation and anaesthetic [44,45,46], and indirectly through productivity losses, all whilst adding a burden to the healthcare system. In the UK, the direct cost of treating complications of non-union is in the range of GBP 7000 to 79,000 [47], with indirect cost through productivity losses estimated to be 10 times this figure in Europe [48].

Our study has also highlighted several factors to be associated with deep infection in subtrochanteric fractures. These include open fractures, the need for massive transfusion and post-operative transfusion. These risk factors are similar to those reported in the literature. The incidence of deep infection has been reported to be as high as 27% in open fractures [49,50]. Although not focused on subtrochanteric fractures, studies have found that transfusion of blood products, in particular massive transfusion, was found to increase the risk of post-operative bacterial infection significantly [51,52]. The increased risk of infection following transfusion could potentially be explained by the immunosuppression known to be associated with transfusion of allogenic blood products [52]; or by the injury patterns (e.g., open fractures, polytrauma) and prolonged surgery necessitating blood transfusion at the first place—all of which expose the host to further increased risk of infection. Furthermore, our studies also found patients with non-union and prolonged LOS to be associated with developing deep infection at the fracture site. Deep infection is most probably the aetiological cause for fracture non-union, as opposed to being caused by it. Similarly, prolonged LOS most likely occurred as a result of patients requiring further interventions and escalation to higher levels of care such as ICU/ HDU to successfully treat their fractures and other associated active medical problems such as systemic infections (HAP, UTI) and polytrauma. Taken altogether, our study and those of others further highlight the multifactorial nature of SSIs, and the importance of identifying modifiable risk factors, and optimise those patients who are at higher risk of developing SSIs.

Our study is the largest published series to date investigating SSIs in subtrochanteric femur fractures which sheds light onto the incidence, causative organisms, treatment outcomes and associations with this devastating complication. Moreover, the diagnosis of SSI in our Trauma Centre was based upon the universally accepted definition set by the CDC and WHO. This, when combined with clinical, serological, imaging and microbiological diagnosis, ensured the uniformity and accuracy of the diagnosis of SSI. However, our study is not without its limitations. Although there are clear criteria for diagnosing superficial infection, each individual’s threshold is still variable and influenced by the level of exposure and experience. This is particularly the case since superficial infection could be diagnosed in the hospital or community, which could be a junior doctor, general practitioner, consultant or a district nurse. Furthermore, the microbiology swab samples, when taken from surgical incision of patients, could possibly be contaminated by normal flora, thereby limiting their efficacy in diagnosing cases suspected of superficial infection. In terms of the accuracy of diagnosis of deep infections, the criterion of three or more positive deep tissue samples has its weakness in its low sensitivity. Techniques such as qPCR would therefore further increase both sensitivity and specificity. This, however, has not found common use in day-to-day microbiological practice. Studies evaluating the cost efficiency of adding qPCR as part of routine investigation is therefore warranted and could potentially improve the accuracy of diagnosis, allow early targeted treatment, improve patient outcomes and therefore improve healthcare spending.

## 6. Conclusions

Causative micro-organisms identified in both superficial and deep infection were similar to those reported in post-trauma osteomyelitis. In an attempt to minimise infection, the treating clinician should focus on modifiable risk factors with adequate patient optimisation, prompt surgical treatment, adequate antibiotic coverage and wound care when treating patients with subtrochanteric femur fracture.

## Figures and Tables

**Table 1:** Patient demographics and characteristics categorised according to patients with no infection, superficial infection and deep infection.

**Demographics**	**No Infection**	**Superficial Infection**	**Deep Infection**
Total number	525	21	15
Age (years)	73.50 (19.07)	68.13 (20.01)	65.83 (19.36)
Gender			
Male	206 (39.2%)	9 (42.9%)	5 (33.3%)
Female	319 (60.8%)	12 (57.1%)	10 (66.7%)
**Injury Characteristics**	**No Infection**	**Superficial Infection**	**Deep Infection**
Mechanism of Injury			
Low energy	411 (78.3%)	13 (61.9%)	9 (60.0%)
High energy	81 (15.4%)	5 (23.8%)	4 (26.7%)
Pathological	33 (6.3%)	3 (14.3%)	2 (13.3%)
Isolated	445 (84.8%)	20 (95.2%)	11 (73.3%)
ISS > 16	33 (6.3%)	0 (0.0%)	3 (20.0%)
Open fracture	5 (1.0%)	0 (0.0%)	2 (13.3%)
**Fracture Characteristics**	**No Infection**	**Superficial Infection**	**Deep Infection**
Russell Taylor Classification			
1A	161 (30.8%)	9 (42.9%)	4 (26.7%)
1B	160 (30.6%)	4 (19.0%)	4 (26.7%)
2A	26 (5%)	1 (4.8%)	0 (0.0%)
2B	176 (33.7%)	7 (33.3%)	7 (46.7%)
Number of fragments (comminution)			
Simple	153 (29.3%)	7 (33.3%)	3 (20.0%)
Moderate	260 (49.7%)	5 (23.8%)	8 (53.3%)
Severe	220 (21%)	9 (42.9%)	4 (26.7%)
Distal extension	173 (33.1%)	3 (14.3%)	10 (66.7%)
**Medical Comorbidities**	**No Infection**	**Superficial Infection**	**Deep Infection**
ASA			
1	45 (8.6%)	3 (14.3%)	1 (6.7%)
2	132 (25.1%)	7 (33.3%)	6 (40.0%)
3	264 (50.3%)	8 (38.1%)	6 (40.0%)
4	84 (16%)	3 (14.3%)	2 (13.3%)
Charlson Comorbidity Score	5.3 (3.1)	4.9 (3.4)	4.7 (3.6)
Diabetes	71 (13.5%)	3 (14.3%)	3 (20.0%)
Steroids	26 (5%)	2 (9.5%)	1 (6.7%)
Malignancy	127 (24.2%)	6 (28.6%)	4 (26.7%)
Dementia	121 (23%)	2 (9.5%)	2 (13.3%)
**Social History**	**No Infection**	**Superficial Infection**	**Deep Infection**
Smoking	127 (24.2%)	6 (28.6%)	6 (40.0%)
Alcohol >10 units/week	97 (18.5%)	2 (9.5%)	6 (40.0%)
Pre-operative Mobility			
Independent	269 (51.2%)	14 (66.7%)	10 (66.7%)
Stick(s)/Crutch(es)	142 (27%)	2 (9.5%)	2 (13.3%)
Frame	91 (17.3%)	2 (9.5%)	2 (13.3%)
Wheelchai/Hoisted	23 (4.4%)	3 (14.3%)	1 (6.7%)
**Operation Characteristics**	**No Infection**	**Superficial Infection**	**Deep Infection**
Operation in less than 48 h	417 (79.4%)	16 (76.2%)	11 (73.3%)
Open reduction	236 (45%)	17 (81.0%)	12 (80.0%)
Use of cerclage wires	57 (10.9%)	2 (9.5%)	3 (20.0%)
Post-op Mobilisation FWB	290 (55.2%)	10 (47.6%)	7 (46.7%)
(first 6 weeks) PWB	114 (21.7%)	3 (14.3%)	5 (33.3%)
TTWB	69 (13.1%)	4 (19%)	0 (0.0%)
NWB	52 (9.9%)	4 (19%)	3 (20.0%)
Surgical time (min) *	110.52 (44.24)	120.67 (42.29)	137.53 (64.43)
Anaesthetic Time (min) **	48.11 (21.3)	50.19 (24.89)	58.27 (26.89)
Level of First Surgeon			
Registrar	308 (59.0%)	16 (76.2%)	8 (53.3%)
Consultant	214 (41%)	5 (23.8%)	7 (46.7%)
Level of Senior Surgeon Present			
Registrar	283 (54.2%)	15 (71.4%)	8 (53.3%)
Consultant	239 (45.8%)	6 (28.6%)	7 (46.7%)
**Complications**	**No Infection**	**Superficial Infection**	**Deep Infection**
Non-union	69 (13.1%)	6 (28.6%)	9 (60.0%)
HAP/CAP	101 (19.2%)	2 (9.5%)	3 (20.0%)
UTI	73 (13.9%)	3 (14.3%)	2 (13.3%)
CKD Stage post-operatively			
Mild	369 (72.2%)	16 (80.0%)	12 (80.0%)
Moderate/Severe	142 (27.8%)	4 (20.0%)	3 (20.0%)
Thromboembolic event			
DVT	9 (7.8%)	3 (33.3%)	0 (0.0%)
PE	10 (8.7%)	0 (0.0%)	0 (0.0%)
Post-operative Transfusion	327 (62.5%)	12 (57.1%)	14 (93.3%)
Transfusion withing 48 h post-operation	264 (50.5%)	11 (52.4%)	13 (86.7%)
Massive transfusion	9 (1.7%)	0 (0.0%)	4 (26.7%)
**Hospital Stay/Mortality**	**No Infection**	**Superficial Infection**	**Deep Infection**
HDU/ICU stay	59 (11.2%)	3 (14.3%)	5 (33.3%)
Total length of hospital stay (days)	21.72 (17.44)	22.86 (16.92)	49.87 (35.38)
Weekend admission	171 (32.6%)	3 (14.3%)	5 (33.3%)
Died within a year	109 (20.8%)	5 (23.8%)	1 (6.7%)

Dichotomous variables are presented as absolute numbers (percentages) of the positive event. Continuous variables are presented as mean (SD). * Surgical time: defined as the time from skin incision to skin closure. ** Anaesthetic time: defined as the time from the start of anaesthetic (induction) to time the patient was positioned in the operating room. ISS: Injury Severity Score; ASA: American Society of Anaesthesiologists Classification; FWB: full weight bearing; PWB: partial weight bearing; TTWB: toe-touch weight bearing; NWB: non-weight bearing; HAP: hospital-acquired pneumonia; CAP: community-acquired pneumonia; UTI: urinary tract infection; CKD: chronic kidney disease; DVT: deep vein thrombosis; PE: pulmonary embolism; HDU: high dependency unit; ICU: intensive care unit.

**Table 2:** Pathogenic micro-organisms for superficial and deep infections. Superficial infections diagnosed according to micro-organisms isolated from the wound swab. Deep infections diagnosed according to micro-organisms isolated from the wound swab and deep tissue samples.

	Superficial Wound Infections		Deep Infection	
	13 Patients *		15 Patients	
Organisms **	*Staphylococcus aureus*	*n* = 6	*Coliforms* ***	*n* = 5
	Enteric flora	*n* = 5	*Staphylococcus aureus*	*n* = 4
	Mixed skin flora	*n* = 5	*Escherichia coli*	*n* = 4
	Enteroccocus species	*n* = 1	*Proteus*	*n* = 3
			*Staphylococcus*	*n* = 1
	Gram-ve bacillus	*n* = 1	*epidermidis*	
	*Beta Haemolytic*	*n* = 1	*Coagulase negative*	*n* = 1
	*Streptococcus Group B*		*Staphylococcus*	
			*Pseudomonas aeruginosa*	*n* = 1
			Gram -ve bacillus	*n* = 1
			*Beta Haemolytic*	*n* = 1
			*Dermabacter*	
			*hominis*	
			No growth	*n* = 3
	Polymicrobial	*n* = 5	Polymicrobial	*n* = 12

* Only 13 of the 21 patients with superficial infection had wound swab. ** More than one organism may have been isolated from each patient. *** Other from *Escherichia coli.*

**Table 3:** Revision operation performed for patients with deep infection.

Operation *	Number of Operations (*n*)
Wound washout, debridement and closure	26
Staged metalwork removal (nail/screws)	9
Exchange nailing	5
RIA debridement of medullary canal	4
Cement nail	3
Local antibiotic therapy (antibiotic beads, antibiotic impregnated cement)	3
Use of Biologic to augment fracture healing (e.g., RIA graft, BMAC, BMP-2) **	2
Blade plate	1
Total hip replacement	1

* Most patients had more than one revision operation (number of revisions: median 3, SD 3.8). ** Commonly performed alongside definitive surgery such as exchange nailing and blade plate.

**Table 4:** Table presenting risk factors associated with deep infection.

**Injury/Fracture Characteristics**	**No Infection**	**Deep Infection**	**Unadjusted OR (95% CI)**	***p*** **-Value**
Open fracture	5 (0.9%)	2 (13.3%)	16.65 (2.95–93.86)	0.001
Distal Extension	176 (32.4%)	10 (66.7%)	4.18 (1.41–12.42)	<0.001
**Social History**	**No infection**	**Deep infection**	**Unadjusted OR (95% CI)**	***p*** **-value**
Alcohol >10 units/week	99 (18.1%)	6 (40.0%)	3.01 (1.05–4.08)	0.041
**Operation Characteristics**	**No infection**	**Deep infection**	**Unadjusted OR (95% CI)**	***p*** **-value**
Open reduction	253 (46.3%)	12 (80.0%)	1.53 (0.65–2.35)	0.019
Surgical time (min)	110.92 (44.17)	137.53 (64.43)	1.01 (1.00–1.02)	0.026
Time from induction to recovery (min)	177.41 (48.26)	228.73 (77.18)	1.02 (1.01–1.02)	<0.001
**Complications**	**No infection**	**Deep infection**	**Unadjusted OR (95% CI)**	***p*** **-value**
Non-union	75 (13.7%)	9 (60.0%)	9.42 (3.26–27.23)	<0.001
Post-operative Transfusion	339 (62.3%)	14 (93.3%)	8.47 (1.11–64.86)	0.040
Massive Transfusion	9 (1.6%)	4 (26.7%)	21.62 (5.77–80.95)	<0.001
HDU/ICU stay	62 (11.4%)	5 (33.3%)	3.90 (1.29–11.79)	0.029
Total length of hospital stay (days)	21.76 (17.41)	49.87 (35.38)	1.03 (1.02–1.05)	<0.001

Continuous variables are presented as mean (SD). HDU: high dependency unit; ICU: intensive care unit; OR: odds ratio; CI: confidence interval.

**Table 5:** Multivariate models demonstrating associations of development of a deep infection following a subtrochanteric fracture.

	OR	95% CI	*p*-Value
Non-union	9.29	2.56–3.38	<0.001
Open fracture	4.23	3.18–5.61	0.005
Massive Transfusion *	1.42	2.39–8.39	0.003
Post-operative Transfusion	1.40	1.10–1.79	0.042
Total LOS	1.04	1.02–1.06	<0.001

* Defined as: transfusion of ≥10 units of red blood cells (RBC) (equivalent of the total blood volume of an average adult patient) within 24 h; transfusion of >4 units of RBC within 1 h with the anticipation of continued need for transfusion; or replacement of >50% of the total blood volume by blood products within 3 h. OR: odds ratio. CI: confidence interval.

## Abbreviations

BMAC	Bone Marrow Aspirate Concentrate
BMP-2	Bone Morphogenetic Protein-2
CAP	Community Acquired Pneumonia
CDC	Centres for Disease Control and Prevention
CKD	Chronic Kidney Disease
CI	Confidence Interval
CRP	C Reactive Protein
DVT	Deep Vein Thrombosis
ESR	Erythrocyte Sedimentation Rate
FBC	Full Blood Count
HAP	Hospital Acquired Pneumonia
HDU	High Dependency Unit
ICU	Intensive Care Unit
IM nail	IntraMedullary Nail
IQr	InterQuartile range
LOS	Length Of Stay
MRI	Magnetic Resonance Imaging
OR	Odds Ratio
PE	Pulmonary Embolism
qPCR	Quantitative Polymerase Chain Reaction
RIA	Reamer-Irrigator-Aspirator
SIRS	Systemic Inflammatory Response Syndrome
SSI	Surgical Site Infection
U&E	Urea and Electrolytes
UTI	Urinary Tract Infection
WHO	World Health Organisation
